# Low-Dose Dioxin Reduced Glucose Uptake in C2C12 Myocytes: The Role of Mitochondrial Oxidative Stress and Insulin-Dependent Calcium Mobilization

**DOI:** 10.3390/antiox11112109

**Published:** 2022-10-26

**Authors:** Suyeol Im, Sora Kang, Ji Hwan Kim, Seung Jun Oh, Youngmi Kim Pak

**Affiliations:** 1 Department of Biomedical Sciences, Graduate School, Kyung Hee University, Seoul 02447, Korea; 2 Department of Neuroscience, Graduate School, Kyung Hee University, Seoul 02447, Korea; 3 Department of Physiology, School of Medicine, Biomedical Science Institute CRI, Kyung Hee University, Seoul 02447, Korea

**Keywords:** environmental toxins, muscle glucose uptake, mitochondrial dysfunction, insulin signal transduction, oxidative stress

## Abstract

Chronic exposure to some environmental polluting chemicals (EPCs) is strongly associated with metabolic syndrome, and insulin resistance is a major biochemical abnormality in the skeletal muscle in patients with metabolic syndrome. However, the causal relationship is inconsistent and little is known about how EPCs affect the insulin signaling cascade in skeletal muscle. Here, we investigated whether exposure to 100 pM of 2,3,7,8-tetrachlorodibenzodioxin (TCDD) as a low dose of dioxin induces insulin resistance in C2C12 myocytes. The treatment with TCDD inhibited the insulin-stimulated glucose uptake and translocation of glucose transporter 4 (GLUT4). The low-dose TCDD reduced the expression of insulin receptor β (IRβ) and insulin receptor substrate (IRS)-1 without affecting the phosphorylation of Akt. The TCDD impaired mitochondrial activities, leading to reactive oxygen species (ROS) production and the blockage of insulin-induced Ca^2+^ release. All TCDD-mediated effects related to insulin resistance were still observed in aryl hydrocarbon receptor (AhR)-deficient myocytes and prevented by MitoTEMPO, a mitochondria-targeting ROS scavenger. These results suggest that low-dose TCDD stress may induce muscle insulin resistance AhR-independently and that mitochondrial oxidative stress is a novel therapeutic target for dioxin-induced insulin resistance.

## 1. Introduction

A number of epidemiologic studies have shown that exposure to various environment-polluting chemicals (EPCs) is strongly associated with the incidence of metabolic diseases, including metabolic syndrome (MetS) [1,2,3]. Among these EPCs, persistent organic pollutants (POPs) are lipophilic and resistant to environmental degradation, so they remain in the environment for many years and bioaccumulate in human adipose tissue. Chronic exposure to some POPs is related with a variety of human diseases such as obesity [4,5], type 2 diabetes (T2D) [3,6,7], and insulin resistance [8,9]. Thus, many EPCs have been listed as endocrine-disrupting chemicals (EDCs) [10], metabolism-disrupting chemicals (MDCs) [11], obesogens, and diabetogens. However, the efforts to establish the cause–effect relationship between exposure to these chemicals and the development of obesity or diabetes in humans have been inconsistent [6]. Recently, we reported that the serum levels of dioxin-like compounds, aryl hydrocarbon receptor (AhR) ligands (AhRL), and mitochondria-inhibiting substances (MIS) were higher in subjects with T2D or insulin resistance than in normal subjects [12]. In addition, the normal subjects with high serum AhRL concentrations had a 7.6-fold higher risk of developing T2D than the subjects with low serum levels of AhR ligands, suggesting that AhR ligands may play an important role in developing these diseases.

MetS is a clinical state in which cardiometabolic risk factors such as abdominal obesity, hyperglycemia, hypertension, and dyslipidemia cluster in one person [13]. Insulin resistance has long been considered a major biochemical abnormality of MetS, as most MetS subjects exhibit impaired insulin action. Insulin resistance generally refers to a state where the tissues do not respond sufficiently to physiological concentrations of insulin [14]. Although the causes and mechanisms leading to insulin resistance have not yet been clearly understood, the poor insulin sensitivity may result from the blockade of the insulin signaling cascade in insulin target tissues such as the skeletal muscle, liver, and white adipose tissue in MetS subjects [15]. The canonical insulin-triggered signaling cascade is initiated by insulin binding to the insulin receptor (IR), and the activated IR recruits and phosphorylates the insulin receptor substrate-1 (IRS-1) on multiple tyrosine residues. Tyrosine-phosphorylated IRS-1 recruits the p85 subunit of phosphatidylinositol 3-kinase (PI3K) to produce phosphoinositide 3,4,5-triphosphate (PIP_3_). Akt is then activated via the phosphorylation of Ser473 and Thr308 by phosphoinositide-dependent kinase (PDK) to initiate Akt-mediated signaling. The insulin signaling ramifies from Akt into diverse functional pathways such as glucose uptake, glycogen synthesis, protein synthesis, lipogenesis, lipolysis, and gluconeogenesis.

The defective insulin signaling is possibly caused by the increased plasma free fatty acid or decreased mitochondrial β-oxidation levels commonly observed in subjects with insulin resistance [16,17]. Increased fatty acid transport to the muscles or decreased intracellular metabolism of fatty acids leads to an increase in intracellular fatty acid metabolites such as diacylglycerol, fatty acyl CoA, and ceramides [18]. These metabolites activate a serine/threonine kinase cascade that induces the phosphorylation of serine/threonine sites on IRS-1. When phosphorylated at the serine/threonine sites, the IRS protein can antagonize tyrosine phosphorylation, inhibiting the canonical insulin signaling. On the other hand, exposure to EPCs may induce mitochondrial dysfunction and oxidative stress [19,20,21]. In human studies, plasma EPC concentrations were positively correlated with lipid peroxidation in the serum [22] or adipose tissue [23]. Multiple EPCs have been shown to impair insulin signaling pathways [24]. For example, 2,3,7,8-tetrachlorodibenzodioxin (TCDD), one of the AhR ligands, inhibited insulin-induced glucose uptake in adipocytes, although the molecular mechanism remains to be resolved [9].

Because skeletal muscle, the largest tissue in the body, requires a lot of energy for locomotion, it is primarily important for systemic insulin sensitivity. The muscles of insulin-resistant subjects typically exhibit reduced muscle mitochondrial capacity [25,26], whereas inherited or acquired mitochondrial abnormalities in human skeletal muscle result in insulin resistance and T2D [26]. In skeletal muscle, the IR-IRS-1-PI3K-Akt activation is an essential step for insulin-induced glucose transporter 4 (GLUT4) translocation onto the cell surface, leading to glucose uptake from the blood stream. In addition to the well-known canonical insulin-triggered signaling cascade of phosphorylation, the insulin-dependent Ca^2+^ mobilization has also been reported to increase GLUT4 translocation [27,28]. In myocytes, insulin induces a fast and transient increase in cytoplasmic Ca^2+^ through L-type Ca^2+^ channels (voltage-dependent calcium channel) activation. Then, a relatively slower Ca^2+^ release rate through the ryanodine receptor (RyR) and inositol 1,4,5-triphosphate receptor (IP_3_R) activation on the sarcoplasmic reticulum (SR) is required for GLUT4 translocation and glucose uptake. This insulin-dependent Ca^2+^ release through RyR or IP_3_R also promotes mitochondrial Ca^2+^ uptake [29]. These actions of insulin on intracellular Ca^2+^ channel activation influence GLUT4 traffic in muscle cells, as well as having other implications for insulin-dependent Ca^2+^ release from the SR. However, little is known about how EPCs affect the insulin signaling cascade and insulin-dependent Ca^2+^ release in skeletal muscle.

In the present study, we investigated whether exposure to low concentrations of TCDD at 100 pM induces insulin resistance in myocytes; 100 pM of TCDD was the mean concentration of serum AhR ligands calculated from T2D and insulin resistance subjects in Korea [12]. We found that this low-dose TCDD stress led to blocked glucose uptake without affecting Akt phosphorylation in myocytes. The TCDD led to enhanced mitochondrial oxidative stress, leading to a decrease in insulin-triggered Ca^2+^ release from the intracellular compartments, which were AhR-independent and reversed by mitochondrial the ROS scavenger, MitoTEMPO.

## 2. Materials and Methods

### 2.1. Cell Culture and Treatment

C2C12 murine myoblasts (CRL-1772, American Type Culture Collection, Manassas, VA, USA) were cultured in Dulbecco’s modified Eagle’s medium (DMEM) containing 25 mM glucose and 1 mM pyruvate under 5% CO_2_ and humidified conditions. The DMEM was supplemented with 10% fetal bovine serum (FBS) and 1% penicillin–streptomycin. The myoblasts were incubated with TCDD (Supelco Inc., Bellefonte, PA, USA) at concentrations of 0.1, 1, or 10 nM after serum starvation for 16 h in serum-deficient media (SDM, DMEM containing 0.5% charcoal-stripped FBS) [30]. For insulin stimulation, the myoblasts were cultured in low-glucose serum-free DMEM containing 5 mM glucose for 3 h and stimulated with 100 nM of insulin (Sigma-Aldrich, St. Louis, MO, USA) for 15 min.

### 2.2. Plasmids and Transfection

The mammalian expression plasmids of pLenti-myc-GLUT4-mCherry (Addgene plasmid # 64049, Watertown, MA, USA), pCMV-mito-R-GECO1-Neo^+^ (Addgene plasmid # 46021), and pcDNA-D1ER-Neo^+^ (Addgene plasmid # 36325) were purchased. The NheI-myc-GLUT4-mCherry-KpnI fragment was amplified by PCR from pLenti-myc-GLUT4-mCherry (5′-TTCGAAGCTAGCGCTACC-3′ and 5′-CGAATTGGTACCAACAAC-3′) and cloned into pcDNA3.1-Neo^+^ (Invitrogen, Carlsbad, CA, USA) to obtain pcDNA3.1-myc-GLUT4-mCherry-Neo^+^.

Double-stranded DNA oligonucleotides for short-hairpin RNA against mouse AhR (shAhR) and 5′-gatccg ACTCTCTGTTCTTAGGCTC ttctcgaga GAGCCTAAGAACAGAGAGT tttttggaag-3′ were cloned into the pSIREN-RetroQ-Puro^+^ vector (Clontech Laboratories, Mountain View, CA, USA). Scrambled control shRNA (shSCR, 5′-gatccg AATTCTCCGAGCGTGTCACGT ttctcgaga ACGTGACACGCTCGGAGAATT tttttggaag-3′) was also produced. The sequence verification was performed via DNA sequencing and NCBI blast searches.

The C2C12 myoblasts were transfected with indicated plasmids using Lipofectamine^®^ 2000 (Invitrogen) according to the manufacturer’s protocol. The stably transfected colonies were selected using 800 μg/mL G418 for mammalian expression plasmids or 1 μg/mL puromycin for pSIREN-RetroQ-shSCR/-shAhR for more than two weeks.

### 2.3. Enzymatic Assay for 2-Deoxyglucose Uptake

To check the glucose uptake, an enzymatic 2-deoxy-D-glucose (2-DG, Sigma-Aldrich) uptake assay was performed with some modifications [31]. Briefly, the reagent-treated myoblasts on 6-well plates were incubated in glucose-free Hank’s Balanced Salt Solution (HBSS: 20 mM HEPES pH 7.4, 145 mM NaCl, 5 mM KCl, 1 mM MgCl_2_, 2 mM CaCl_2_) for 2 h, stimulated by 100 nM of insulin for 10 min, and incubated with 1 mM 2-DG for 30 min at 37 °C. The harvested cells were resuspended in 20 mM Tris-HCl (pH 8.8) and disrupted via sonication. The endogenous NADP^+^ was decomposed via incubation at 85 °C for 60 min. The proteins were precipitated by 0.6 M perchloric acid and then the samples were centrifuged at 16,000× *g* for 10 min in 4 °C. The supernatants were neutralized via the addition of 2 M KHCO_3_ and further centrifuged at 16,000× *g* for 10 min at 4 °C. The supernatants were incubated with assay solution (final concentration: 50 mM triethanolamine hydrochloride (pH 8.0), 1 mM ATP, 50 mM KCl, 10 μM NADP^+^, 1 mM MgCl_2_, 10 units/mL glucose-6-phosphate dehydrogenase, 1 unit/mL diaphorase, 5 units/mL hexokinase, 0.01% bovine serum albumin (BSA), and 20 μM resazurin) in 37 °C for 3 h and the fluorescence was measured at 535/587 nm using a spectrofluorometer (SpectraMax^®^ Gemini™ EM, Molecular Devices, Sunnyvale, CA, USA). The amounts of 2-DG transported into the cells were calculated from a standard curve and normalized by protein concentration.

### 2.4. Assays for GLUT4 Translocation

The translocation of GLUT4 from the GLUT4 vesicles to the plasma membrane was monitored via two different methods using myc-GLUT4-mCherry-transfected cells. The C2C12 cells were transfected with pcDNA3.1-myc-GLUT4-mCherry and the stable cells were selected using 800 μg/mL G418 for 2 weeks. To monitor the translocation of the myc-GLUT4-mCherry fusion protein under a confocal microscope, the C2C12 cells expressing myc-GLUT4-mCherry were fixed in 2% paraformaldehyde for 10 min at room temperature and immunostained in a non-permeabilized condition [32]. Then, the cells were incubated with primary mouse anti-myc antibody (1:250, 9E10, Santa Cruz Biotech., Dallas, TX, USA) overnight at 4 °C and incubated with Alexa Fluor 488-conjugated anti-mouse IgG antibody for 1 h at room temperature. The mounted samples were subjected to confocal imaging. The confocal images were acquired using an LSM700 laser-scanning confocal microscope (Carl Zeiss, Oberkochen, Germany), using a 488 nm laser for the myc and 555 nm laser for the mCherry.

The surface myc-GLUT4 level was also quantified by colorimetric assay using *o*-phenylenediamine dihydrochloride (OPD, Cat# P8287, Sigma-Aldrich) [29,33]. OPD is a water-soluble substrate for horseradish peroxidase (HRP) that produces a yellow-orange product detectable at 450 nm by ELISA plate readers. The C2C12 cells expressing myc-GLUT4-mCherry were fixed with 2% paraformaldehyde for 10 min at room temperature. The cells were blocked with 3% BSA in PBS for 30 min and incubated with anti-myc antibody (1:250, 9E10, Santa Cruz Biotech) at 4 °C overnight. The cells were extensively washed with PBS and further incubated with anti-mouse IgG antibody conjugated with peroxidase for 1 h at room temperature. The cells were thoroughly washed with PBS and then the OPD reagent (200 μL/well) was added. The OPD reagent (1 mg/mL) containing hydrogen peroxide (4 μL of 30% H_2_O_2_ in 10 mL solution) was freshly prepared in 50 mM of phosphate–citrate buffer (pH 5.0). After the reaction performed in the dark for 30 min at room temperature, the absorbance of the supernatant was measured at 450 nm using a Versamax microplate reader (Molecular Devices).

### 2.5. Western Blot

The cell lysates were prepared on ice in PRO-PREP Protein Extraction Solution (iNtRON Biotechnology, Gyeonggi-do, Korea) containing a protease/phosphatase inhibitor cocktail (GeneDEPOT, Barker, TX, USA). The protein concentration was determined using a Pierce™ BCA Protein Assay Kit (Thermo Fisher, Waltham, MA, USA). The cell lysates (30 μg) were separated on 8–15% SDS–polyacrylamide gel and transferred to the PVDF membrane. The membranes were incubated with appropriate primary antibodies in Tris-buffered saline with 0.1% Tween 20 (TBST) containing 3% BSA. The sources and working dilutions of the antibodies are summarized in Supplementary Table S1. The bands on the PVDF membrane were detected with G:BOX Chemi XL1.4 (Syngene, Frederick, MD, USA) using EzWestLumi plus (ATTO, Tokyo, Japan) and the band intensities were quantified using the ImageJ program. All uncropped scans of Western blots are shown in the Supplementary Information.

### 2.6. Real-Time Quantitative RT-PCR

The total RNA was isolated with the FavorPrep™ Tri-RNA reagent (Favorgen Biotech Corp, Ping-Tung, Taiwan). The total RNA (1.5 μg) was then reverse transcribed using MMLV Reverse Transcriptase (Promega, Madison, WI, USA) and RNasin Ribonuclease inhibitors (Promega) with 10 ng random hexamers (Invitrogen) and 25 mM of dNTP mix (Gene Craft, Ludinghausen, Germany), according to the manufacturer’s instructions. The SYBR Green dye-based real-time qRT-PCR was performed using the AMPIGENE^®^ Taq Mix (Enzo Life Sciences, Inc., Farmingdale, NY, USA) on Rotor-Gene Q (QIAGEN, Germantown, MD, USA). The primers used in the real-time qRT-PCR are described in Table 1. The measurements were performed in triplicate for each sample. The quantity of the mRNA was corrected via simultaneous measurements of the nuclear DNA encoding the 18S rRNA. The relative gene expression level was determined using the 2^–ΔΔCt^ method [34]. The relative mRNA expression level is presented as the fold change compared to that of the control condition.

### 2.7. Assays for Mitochondrial Activity

The mitochondrial membrane potential (ΔΨ_m_) was determined by loading the cells with tetramethylrhodamine ethyl ester (TMRE, Invitrogen). Briefly, the C2C12 myoblasts were stained with 1 μM TMRE for 30 min at 37 °C and harvested after washing with DPBS twice. The TMRE-mediated fluorescence levels of over 12,000 cells were analyzed using a flow cytometer (Gallios, Beckman Coulter, Inc., Brea, CA, USA).

The intracellular ATP level was determined using a CellTiter-Glo^®^ luminescence-based assay kit (Promega). The cells cultured on a 96-well white plate (Corning Life Science, Oneonta, NY, USA) were incubated with TCDD and washed with DPBS. The luminescence was measured using a Centro LB 960 microplate luminometer (Berthold, Bad Wildbad, Germany).

To detect the generation of cytosolic and mitochondrial reactive oxygen species (ROS), 5,6-chloromethyl-2′,7′-dichlorodihydrofluorescein diacetate, acetyl ester (CM-H_2_DCFDA, Molecular Probes, Eugene, OR, USA), and MitoSOX™ (Molecular Probes) were used, respectively [35]. The C2C12 myoblasts (1 × 10^4^ cells/well) seeded on a clear-bottom 96-well black plate (Corning Life Science) were incubated with 1 μM of CM-H_2_DCFDA or 5 μM of MitoSOX for 30 min at 37 °C and washed with HBSS. The fluorescence intensity was measured using a spectrofluorometer (SpectraMax^®^, Gemini™ EM, Molecular Device) at a wavelength of 494/522 nm for CM-H_2_DCFDA and 510/580 nm for MitoSOX.

### 2.8. Measurement of Oxygen Consumption Rate

The endogenous cellular oxygen consumption rate (OCR) and extracellular acidification rate (ECAR) were measured in C2C12 cells using a Seahorse XF-24 Analyzer (Agilent Technologies, Santa Clara, CA, USA) following the procedure from the manufacturer [35,36]. During the measurements, 1 μM oligomycin, 2 μM carbonyl cyanide-4-(trifluoromethoxy) phenylhydrazone (FCCP), and 1 μM rotenone/antimycin A were consecutively added to inhibit oxidative phosphorylation (OXPHOS) complexes. The OCR and ECAR were calculated from 3 min measurement cycles and normalized to the cell number. From the OCR profiles, the ATP turnover rate (basal OCR—oligomycin-OCR) and total respiratory capacity (FCCP-OCR—rotenone-OCR) were calculated. The ATP production rates from mitochondria (mitoATP) and glycolysis (glycoATP) were calculated from the OCR and ECAR obtained during the basal conditions [37].

### 2.9. OXPHOS Complex I Activity

An in-gel activity (IGA) assay was performed to check the activity of OXPHOS complex I after the blue native polyacrylamide gel electrophoresis (BN-PAGE) of isolated mitochondria [38]. Briefly, the C2C12 cells (1 × 10^6^ cells/100 mm dishes) were homogenized by passing them through a 23 G needle in mitochondria isolation buffer (0.25 M Tris-HCl pH 7.4 and 1 mM EDTA) containing a protease/phosphatase inhibitor cocktail (GenDEPOT, Barker, TX, USA). The mitochondria were isolated via the differential centrifugation of the homogenate at 1000× *g* for 10 min in 4 °C and 7500× *g* for 10 min in 4 °C. The isolated mitochondria (20 μg protein) were solubilized using 1% *n*-dodecyl-β-D-maltoside (Sigma-Aldrich), mixed with 0.125% Coomassie blue G250, and then separated using 4–16% Blue Native PAGE gel (BN-PAGE, Cat# BN1002BOX, Thermo Fisher) at 4 °C. After the BN-PAGE separation, the gels were incubated with IGA buffer (0.1 M Tris-HCl pH 7.4, 1 mg/mL nitro blue tetrazolium, and 0.14 mM NADH) for 2 h at room temperature. The band intensity indicating the activity of the OXPHOS complex I was normalized to that of HSP60 in the Western blot of the same mitochondria used for the BN-PAGE separation.

### 2.10. Calcium Monitoring

C2C12 myoblasts (1 × 10^4^ cells) cultured on confocal dishes (SPL Life Sciences, Pocheon, Korea) were preloaded with 3 μM of Fluo-4 AM (Invitrogen) in DMEM for 30 min at room temperature, washed with HBSS, and used within 2 h. Cellular calcium images with Fluo-4 fluorescence were collected every second for 5 min using an LSM700 laser-scanning confocal microscope (Carl Zeiss) with a 488 nm laser during the sequential stimulation of insulin (100 nM) and ionomycin (10 μM) [29,39]. The relative fluorescence is the ratio of the fluorescence difference relative to the maximum fluorescence (*F* − *F*_0_)/(*F_max_* − *F*_0_), where *F* is the observed fluorescence, *F*_0_ is the basal fluorescence before insulin stimulation, and *F_max_* is the maximum fluorescence intensity after adding 10 μM of ionomycin Ca^2+^ ionophores. The relative fluorescence is expressed as a function of time.

The ratiometric Ca^2+^ measurement was performed with Fura-2 AM using a spectrofluorometer (SpectraMax^®^ Gemini™ EM, Molecular Devices) and following the manufacturer’s instructions. The fluorescence ratio (340/380 nm) was used to monitor changes in cytosolic Ca^2+^ after the TCDD treatment. C2C12 myoblasts (1 × 10^4^ cells/well) in a 96-well black plate were incubated with TCDD. The cells were loaded with 2 μM Fura-2 AM in HBSS for 20 min at room temperature and washed with HBSS. The fluorescence levels excited at 340 nm and 380 nm and the emitted fluorescence at 505 nm were measured using a spectrofluorometer.

The mitochondrial and ER Ca^2+^ levels were monitored using CMV-mito-R-GECO1 (red intensiometric, genetically encoded Ca^2+^-indicators for the optical imaging of mitochondrial calcium, neomycin-resistant) and pcDNA-D1ER (D1ER second-generation chameleon calcium sensor targeted to ER, neomycin-resistant), respectively. For the mitochondrial Ca^2+^, CMV-mito-R-GECO1-transfected C2C12 cells (1 × 10^4^ cells) were seeded on confocal dishes and incubated with 0.1% DMSO or 100 pM of TCDD for 48 h in SDM. The cells were stained with 200 nM of MitoTrackerTM Green (Invitrogen) for 30 min to check the mitochondrial morphology and then the signals were acquired using a LSM700 confocal microscope. For the ER Ca^2+^, pcDNA-D1ER-transfected C2C12 cells (1 × 10^4^ cells) were cultured on a black 96-well plate. The fluorescence intensity levels were measured at 435/475 nm and 435/575 nm (ex/em) using a spectrofluorometer (SpectraMax^®^) and the ratio was calculated.

### 2.11. Measurement of Glucose Uptake in Mouse Muscles

Seven-week-old C57BL6 mice (*n* = 6/group) were purchased from Daehan BioLink (Eumseong, Korea) and maintained for 1 week before experiment. The amounts of 2-DG uptake in whole muscles were measured essentially as described [40]. The mice were intraperitoneally (i.p.) injected with 10 μg/kg of TCDD with or without 10 μg/kg of MitoTEMPO ([2-[(1-hydroxy-2,2,6,6-tetramethylpiperidin-4-yl)amino]-2-oxoethyl]-triphenylphosphanium chloride, Sigma-Aldrich). Two weeks later, 1 mg/kg of insulin was i.p.-injected into the mice 10 min before the i.p. injection of 3 mmol/kg of 2-DG. The extensor digitorum longus (EDL, slow-twitch, white muscle) and soleus (fast-twitch, red muscle) muscles were dissected 30 min after the 2-DG injection and immediately frozen in liquid nitrogen. The frozen mouse tissues were homogenized in 20 mM of Tris-HCl (pH 8.8). After heating the homogenized lysates at 85 °C for 60 min, followed by centrifugation at 16,000× *g* for 20 min at 4 °C, the supernatants were used to measure the 2-DG uptake as described in Section 2.3. All procedures for handling mice were carried out in accordance with the Principles of Laboratory Animal Care (NIH publication No. 85–23, revised 1985) and the Animal Care and Use Guidelines (KHSASP-20-163) of Kyung Hee University, Seoul, Korea.

### 2.12. Statistical Analysis

The data values are expressed as the means ± standard error of the mean (SEM). The statistical analysis was performed using Graph Pad Prism software. The statistical significance between groups was evaluated using an unpaired Student’s *t*-test. Significance was defined by a *p* value < 0.05.

## 3. Results

### 3.1. Inhibition of Insulin-Triggered Glucose Uptake by TCDD

**Figure 1:**
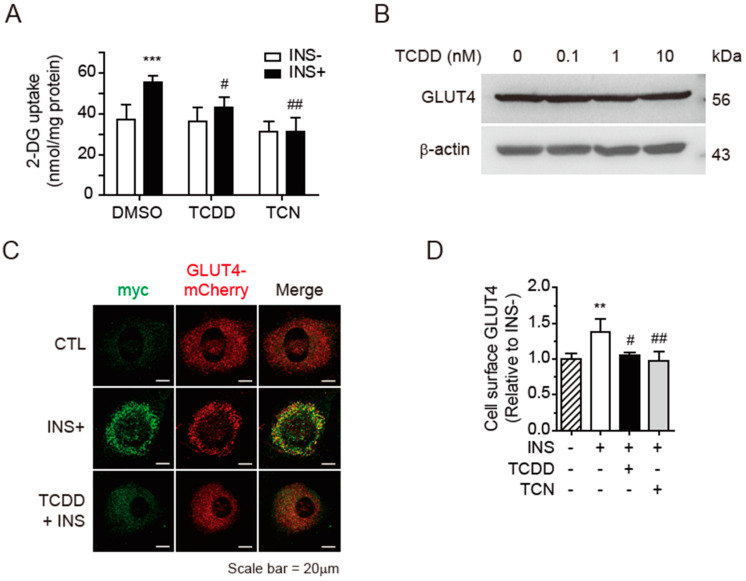
TCDD suppressed the insulin-stimulated glucose uptake and GLUT4 translocation. C2C12 myoblasts were treated with DMSO, 100 pM of TCDD, or 2 μM of triciribine (TCN) for 48 h and stimulated with 100 nM of insulin (INS+) for 10 min. (**A**) Glucose uptake. The reagent-treated cells were incubated with 1 mM of 2-deoxyglucose (2-DG) for 30 min. The amounts of 2-DG transported into the cells were measured via enzymatic assay for 2-DG uptake. (**B**) Western blot. TCDD-treated C2C12 cell lysates were separated on 10% SDS-PAGE and detected with GLUT4 antibody, with β-actin as the loading control. (**C**) Representative confocal image of the subcellular colocalization of myc (green) with GLUT4-mCherry (red). The C2C12 cells expressing myc-GLUT4-mCherry were incubated with DMSO or 100 pM of TCDD for 48 h with or without stimulation with insulin (INS+). The cells were fixed and incubated with mouse anti-myc antibody and Alexa Fluor 488-conjugated secondary antibody under non-permeabilized conditions. Confocal images were obtained at 488 or 555 nm for myc or GLUT4-mCherry, respectively. Scale bar = 20 μm. (**D**) Colorimetric quantification of cell surface myc (GLUT4) using *o*-phenylenediamine dihydrochloride reagent. Data are presented as means ± SEM (*n* = 4~6). Note: ** *p* < 0.01, *** *p* < 0.001 vs. INS- control; ^#^ *p* < 0.05, ^##^ *p* < 0.01 vs. INS+ control.

To test whether a low concentration of TCDD induces insulin resistance in skeletal muscles, the insulin-stimulated glucose uptake was measured in C2C12 myoblasts using an enzymatic assay for 2-DG uptake. Treatment with 100 pM of TCDD for 48 h led to blocked insulin-stimulated 2-DG uptake, as with the Akt inhibitor triciribine (TCN) used as the control (Figure 1A). It has been reported that 10 nM of TCDD decreases the mRNA level of GLUT4 in 3T3-L1 adipocytes, impairing the insulin signaling pathway and leading to insulin resistance [9]. However, the Western blot of GLUT4 protein in the C2C12 cells revealed that the TCDD did not alter the expression of GLUT4, even at 10 nM (Figure 1B). Next, we checked the insulin-stimulated exocytosis of GLUT4 from GLUT4 storage vesicles to the cellular surface using pcDNA3.1-myc-GLUT4-mCherry-transfected C2C12 myoblasts. In the myc-GLUT4-mCherry fusion protein, the myc epitope was inserted in the first exofacial loop of the GLTU4 N-terminus and the mCherry was fused at the C-terminus [41]. Thus, the fusion protein allows the detection of GLUT4 translocation on plasma membrane surfaces using myc in non-permeabilized conditions and of the total GLUT4 content based on the mCherry fluorescence. The insulin stimulation induced significant exposure of the myc epitope (green) to the plasma membrane, but 100 pM of TCDD suppressed the insulin-stimulated myc exposure (Figure 1C). To quantify the cell surface GLUT4 presentation, the amount of OPD precipitate was determined on the plasma membrane after the myc immunoassay (Figure 1D). The TCDD (100 pM) significantly inhibited the insulin-stimulated GLUT4 exocytosis from GLUT4 vesicles to the plasma membrane to a level comparable to that of TCN-treated Akt inhibitor cells.

### 3.2. Low-Dose TCDD Reduced IRβ and IRS-1 Protein Expression without Affecting Akt Phosphorylation

**Figure 2:**
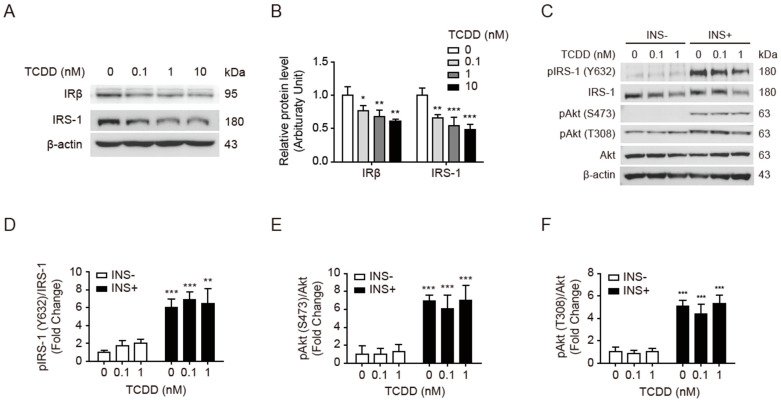
TCDD downregulated IRβ and IRS-1 protein expression without reducing Akt phosphorylation. C2C12 myoblasts were treated with TCDD (0, 0.1, 1, or 10 nM) for 48 h with or without stimulation with 100 nM of insulin (INS+) for 15 min. (**A**,**C**) Western blot of insulin signaling proteins. TCDD-treated C2C12 cell lysates were separated on 10% SDS-PAGE and detected with the indicated antibodies; β-actin is a loading control. (**B**) Quantification of band intensities of Western blot of panel A. (**D**–**F**) Quantification of band intensities of Western blot of panel C. The ratios of pIRS-1 and pAkt to IRS-1 and Akt are presented as fold changes relative to the DMSO-treated control. Data are presented as means ± SEM (*n* = 4). Note: * *p* < 0.05, ** *p* < 0.01, *** *p* < 0.001 vs. DMSO or INS-control.

We studied whether 100 pM of TCDD affects the expression of insulin signaling molecules in skeletal muscle. As reported in adipocytes [9], the TCDD decreased the levels of insulin receptor β (IRβ) and IRS-1 proteins in a dose-dependent manner (Figure 2A,B). However, the S307 phosphorylation of IRS-1, a hallmark of insulin resistance, was also decreased by the TCDD rather than increased in unstimulated cells (Figure 2A). In myocytes, low-dose TCDD might not affect the kinase activity for these signaling molecules. The insulin-stimulated phosphorylation of IRS-1(Y632) was decreased along with the expression level of IRS-1 (Figure 2C,D). Notably, the phosphorylated Akt, pAkt (S473), and pAkt (T308)-to-Akt ratios were maintained in both unstimulated and insulin-stimulated myocytes, despite the decreased IRβ and IRS-1 protein levels (Figure 2C,E,F). This suggests that mechanisms other than Akt-mediated insulin signaling may be involved in insulin resistance induced by TCDD exposure.

### 3.3. TCDD Impaired Mitochondrial Activity

**Figure 3:**
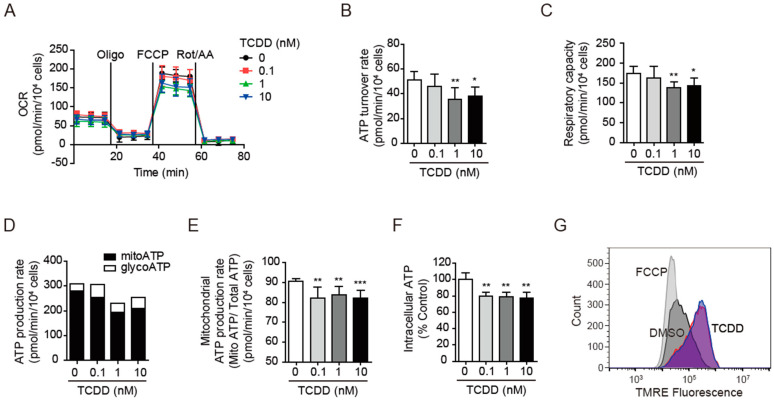
Impairment of mitochondrial activities by TCDD. (**A**) Oxygen consumption rate (OCR) profiles. C2C12 cells on an XF-24 microplate were pre-treated with TCDD (0, 0.1, 1, or 10 nM) for 48 h and OCRs were analyzed using a Seahorse XF-24 analyzer. Oligomycin (Oligo), FCCP, and rotenone/antimycin A (Rot/AA) were consecutively injected to obtain mitochondrial respiration capacities. (**B**) ATP turnover rate (basal OCR—oligomycin-inhibited OCR). (**C**) Respiratory capacity (FCCP-induced OCR). (**D**) ATP production rates from mitochondria (mitoATP, black bar) and glycolysis (glycoATP, white bar) were calculated from the OCR and ECAR. (**E**) Mitochondrial ATP production ratio. Ratio of mitoATP to total ATP. (**F**) Intracellular ATP contents in TCDD-treated cells were determined using CellTiter-Glo^®^. Data are graphed as means ± SEM (*n* = 4~6). Note: * *p* < 0.05, ** *p* < 0.01, *** *p* < 0.001 vs. DMSO-treated cells. (**G**) Flow cytometry histogram. The TMRE-mediated mitochondrial membrane potential was measured via flow cytometry. C2C12 cells were treated with 100 pM (red) or 10 nM (blue) TCDD for 48 h and stained with 1 μM TMRE for 30 min, and the fluorescence levels of over 12,000 cells were analyzed. Cells treated with FCCP (10 μM, white gray) for 1 h were used as the positive control.

The mitochondrial function interacts bidirectionally with insulin sensitivity depending on the tissue [42]. Reduced mitochondrial activity was observed in human insulin-resistant skeletal muscle [17], and conversely abnormal mitochondrial function resulted in insulin resistance and T2D [43]. To determine whether 100 pM of TCDD (low-dose TCDD) induces mitochondrial abnormalities in C2C12 cells and thereby leads to reduced insulin-stimulated glucose uptake and insulin signaling, we analyzed the mitochondrial activities in various aspects. When measuring the OCR profiles in C2C12 cells, the TCDD slightly decreased the OCR profiles (Figure 3A), ATP turnover rate (Figure 3B), and respiratory capacity (Figure 3C) in a dose-dependent manner, but its effects were relatively small compared to the other tissues [30]. When the ATP production rates from mitochondria (mitoATP, black bar) and glycolysis (glycoATP, white bar) were calculated from the OCR and ECAR profiles (Figure 3D), the mitochondrial ATP production ratio (mitoATP/total ATP) (*p* < 0.01, Figure 3E) clearly showed that the TCDD decreased the mitoATP dose-dependently, but not the glycoATP. Similarly, the TCDD reduced the intracellular ATP contents, which were almost saturated with 100 pM of TCDD (Figure 3F). When the TMRE-mediated mitochondrial membrane potential (ΔΨ_m_) was determined using flow cytometry, the 100 pM TCDD-treated C2C12 cells unexpectedly displayed hyperpolarization (more negative) rather than dissipation of ΔΨ_m_ (Figure 3G). Despite the hyperpolarization of ΔΨ_m_, the ECAR was increased in the TCDD-treated cells. The glycolysis stress test using an OCR/ECAR profile analysis revealed that the TCDD treatment altered the cellular status from aerobic to glycolytic (Supplementary Figure S1). Accordingly, the TCDD acidified the cytosolic pH level and significantly increased the mRNA levels of glycolytic enzymes, pyruvate kinase isozyme M2 (PKM2), and lactate dehydrogenase A (LDHA).

### 3.4. TCDD Increased OXPHOS Complex I Activity and Mitochondrial ROS

**Figure 4:**
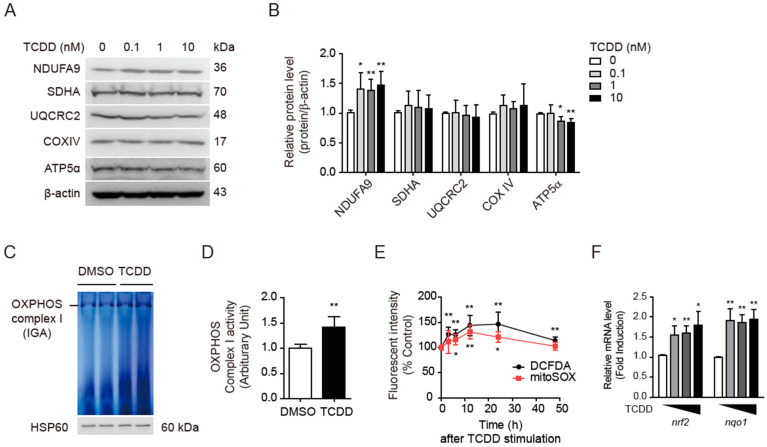
TCDD increased the OXPHOS complex I activity and ROS. C2C12 myoblasts were treated with TCDD (0, 0.1, 1, or 10 nM) for 48 h under unstimulated condition. (**A**) Western blot of subunits of OXPHOS complexes. The cell lysates were separated on 10–15% SDS-PAGE and detected with the indicated antibodies of OXPHOS complexes. (**B**) Quantification of band intensities of panel A. (**C**) In-gel activity (IGA) assay of OXPHOS complex I. Mitochondria were isolated from TCDD-treated C2C12 cells, separated by 4–16% Blue Native PAGE gel. The gel was incubated with IGA buffer containing nitro blue tetrazolium and NADH. Western blot of the same mitochondria using HSP60 antibody for the loading control. (**D**) Quantification of band intensities of the IGA assay shown in (**C**). The band intensity ratio of the OXPHOS complex I to HSP60 is shown. (**E**) Time-dependent production of cellular (DCFDA) and mitochondrial (mitoSOX) ROS. C2C12 cells treated with TCDD (100 pM) for designated periods were labeled with 1 μM DCFDA or 5 μM mitoSOX and fluorescence intensity was determined by fluorometer. (**F**) Realtime qRT-PCR. mRNA levels of NRF2 and NQO1 after TCDD treatment for 48 h. Data are graphed as means ± SEM (*n* = 4~5). * *p* < 0.05, ** *p* < 0.01 vs. DMSO control.

The hyperpolarization of ΔΨ_m_ could be induced either by the inhibition of OXPHOS complex I or by the low activity of complexes II, III, and IV [44]. Because the TCDD did not appear to interact directly with the OXPHOS complexes, the expression levels of subunits representing the five OXPHOS complexes were analyzed by Western blot (Figure 4A). TCDD treatment for 48 h led to increased NDUFA9 (NADH dehydrogenase (ubiquinone) 1 α subcomplex subunit 9, complex I) and decreased ATP5α (ATP synthase F1 subunit α, complex V) (*p* < 0.05, Figure 4A,B). However, the expression levels of SDHA (succinate dehydrogenase subunit α, complex II), UQCRC2 (mitochondrial cytochrome b-c1 complex subunit 2, complex III), and COX IV (cytochrome c oxidase subunit IV, complex IV) were not altered. To confirm whether the activity of the OXPHOS complex I was indeed enhanced, a mitochondrial IGA assay was performed in the presence of nitro blue tetrazolium and NADH on Blue Native PAGE gel (Figure 4C). The IGA showed that the 48 h treatment with TCDD (100 pM) significantly increased the activity of OXPHOS complex I in the mitochondria (*p* < 0.01, Figure 4D). Since complexes I and III of OXPHOS are considered the major sources of mitochondrial ROS (mtROS) [45], the TCDD-enhanced activity of complex I could induce the mtROS production. 

However, the DCFDA-probed cellular and mitoSOX-probed mtROS levels were not significantly increased by the 100 pM TCDD treatment for 48 h (Figure 4E). When the time-dependent production of ROS was determined, the 100 pM TCDD treatment increased both the cellular ROS and mtROS by 30~50% within 24 h, then the ROS levels decreased to near-normal levels after 48 h (Figure 4E). The TCDD treatment for 48 h induced antioxidant genes such as NRF2 and NQO1 in a dose-dependent manner (Figure 4F), which is in good agreement with the reports by others [46]. Thus, the normalization of the ROS level is thought to be due to the induction of antioxidant proteins.

### 3.5. TCDD Disturbed the Insulin-Triggered Calcium Release into Cytosol

**Figure 5:**
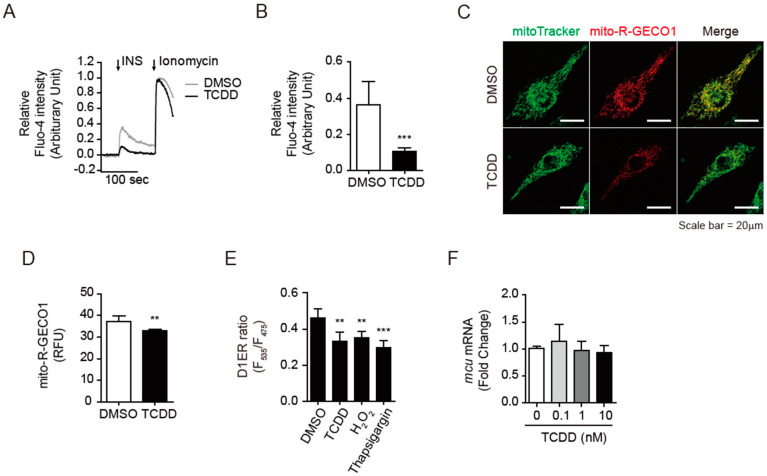
TCDD disturbed the insulin-triggered Ca^2+^ release into the cytosol. C2C12 cells were incubated with DMSO or 100 pM of TCDD for 48 h. (**A**) Time-dependent relative Fluo-4 fluorescence intensity. The cells were loaded with 3 μM of Fluo-4 AM for 30 min and sequentially stimulated by 100 nM of insulin and 10 μM of ionomycin. The fluorescence was monitored by confocal microscope. (**B**) Insulin-induced calcium release. The insulin-stimulated relative fluorescence intensity was calculated from (**A**). (**C**) Confocal images. Mito-R-GECO1-transfected cells were stained with 200 nM MitoTracker™ Green for 30 min. Mitochondria (MitoTracker, green) and mitochondrial calcium (Mito-R GECO1, red) were analyzed using a confocal microscope. Scale bar = 20 μm. (**D**) Mitochondrial calcium level. The fluorescent intensities of Mito-R-GECO1 of the cells was determined. (**E**) ER calcium. Calcium in the ER was measured using a fluorometer in D1ER-transfected cells. To verify the assay, a decrease in ER calcium was induced via incubation with 100 μM of H_2_O_2_ or 5 μg/mL of thapsigargin for 3 h before experiment. (**F**) Real-time qRT-PCR. Relative mRNA level of MCU after TCDD treatment. Data are presented as means ± SEM (*n* = 3~6). Note: ** *p* < 0.01, *** *p* < 0.001 vs. DMSO control.

Since the TCDD treatment did not significantly alter the canonical insulin-triggered signaling cascade through Akt, we explored whether TCDD affected the insulin-dependent Ca^2+^ mobilization in C2C12 cells. In skeletal muscle, insulin can stimulate glucose uptake by activating the RyR and IP_3_R, i.e., the sarco-endoplasmic reticulum (SER) channels that release Ca^2+^ into the cytosol [27]. Insulin-mediated PI3K activation enhances NADPH oxidase (NOX2)-mediated local ROS production to open RyR channels. At the same time, insulin activates IP_3_R to release Ca^2+^ from the SER and to increase mitochondrial Ca^2+^ uptake [29]. To determine the effect of 100 pM of TCDD on the cytosolic Ca^2+^ concentration, C2C12 cells were pre-loaded with Fluo-4 AM and the fluorescence intensity was monitored with and without TCDD pre-treatment using a confocal microscope. In the control cells, stimulation with 100 nM of insulin induced an increase in cytosolic Ca^2+^, whereas the pre-treatment with TCDD abolished the insulin-stimulated Ca^2+^ increase (Figure 5A,B).

Next, the effects of TCDD on mitochondrial or ER Ca^2+^ levels were measured using mito-R-GECO1 or D1ER-transfected C2C12 cells, respectively. The TCDD (100 pM) treatment reduced the mitochondrial Ca^2+^-mediated fluorescence intensity by 12% (*p* < 0.01, Figure 5C,D). The ER Ca^2+^ levels were also decreased by 20%, similar to the controls treated with H_2_O_2_ or thapsigargin, a SER Ca^2+^ ATPase (SERCA) inhibitor (Figure 5E). Ca^2+^ in the SR/ER is transferred into mitochondria through the mitochondrial calcium uniporter (MCU) in the mitochondria-associated membrane (MAM), the junction of the physically connected ER and mitochondria [47]. Although TCDD reduced the mitochondrial Ca^2+^ levels, the mRNA level of the MCU was not significantly altered by the TCDD (Figure 5F), suggesting that the MCU may not be involved in the TCDD-mediated decrease in mitochondrial Ca^2+^, at least at the expression level.

### 3.6. AhR-Independent Effects of TCDD in Skeletal Muscle

**Figure 6:**
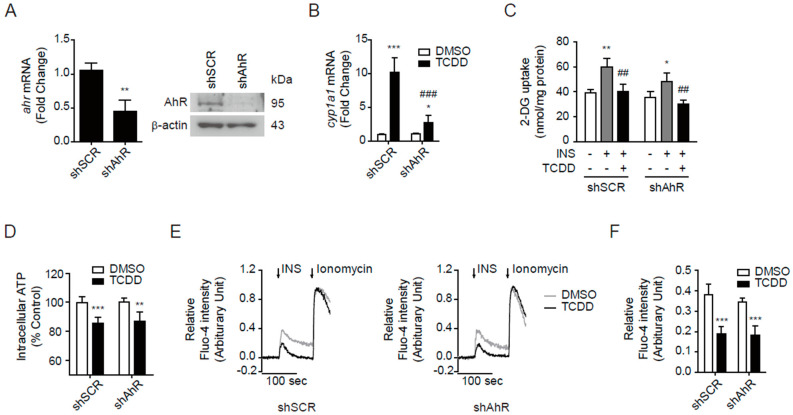
TCDD inhibited insulin-induced glucose uptake in an AhR-independent manner. The shSCR and shAhR C2C12 cells were incubated with DMSO or TCDD (100 pM) for 48 h. (**A**) The mRNA (left panel) and protein (right panel) levels of AhR in shSCR and shAhR cells. (**B**) CYP1A1 induction by TCDD in shSCR and shAhR cells. (**C**) Glucose uptake. The cells were incubated in glucose-free HBSS for 2 h, stimulated by insulin (100 nM, 10 min), and incubated with 2-DG (1 mM, 30 min). The concentration of 2-DG in the cells was measured using an enzymatic assay and normalized to the protein concentration. (**D**) Intracellular ATP contents in TCDD-treated cells were determined using CellTiter-Glo^®^. (**E**) The cells were loaded with 3 μM of Fluo-4 AM for 30 min and sequentially stimulated by 100 nM of insulin and 10 μM of ionomycin. The fluorescence was monitored using a confocal microscope. (**F**) The insulin-stimulated relative fluorescence intensity was calculated from (**E**). Data are represented as means ± SEM (*n* = 3~9). Note: * *p* < 0.05, ** *p* < 0.01, *** *p* < 0.001 vs. DMSO control in shSCR or shAhR cells; ^##^ *p* < 0.01, ^###^ *p* < 0.001 vs. INS+ group in shSCR or shAhR cells or TCDD-treated shSCR cells.

A well-established mechanism of TCDD’s action is AhR–Arnt-mediated transcription activation. Therefore, we examined whether AhR is involved in the effects of low-dose TCDD on the glucose uptake, mitochondrial activity, and Ca^2+^ concentration using stable pSIREN-shAhR plasmid-transfected C2C12 myoblasts. In shAhR-transfected cells, the expression levels of AhR mRNA and protein were reduced by 30~50% (Figure 6A). Additionally, in shAhR cells, the TCDD did not increase the mRNA of CYP1A1, a target gene of AhR, by as much as in the control shSCR cells (Figure 6B). However, TCDD blocked the insulin-stimulated glucose uptake in both shAhR-cells and shSCR-cells (Figure 6C). TCDD also decreased the intracellular ATP contents (Figure 6D) and insulin-stimulated Ca^2+^ release (Figure 6E,F) in the absence of AhR. The results suggest that TCDD induces a dysregulation of glucose uptake, mitochondrial function, and Ca^2+^ homeostasis in C2C12 cells, regardless of the presence of AhR.

### 3.7. Calcium Dysregulation Resulted from Mitochondrial ROS

**Figure 7:**
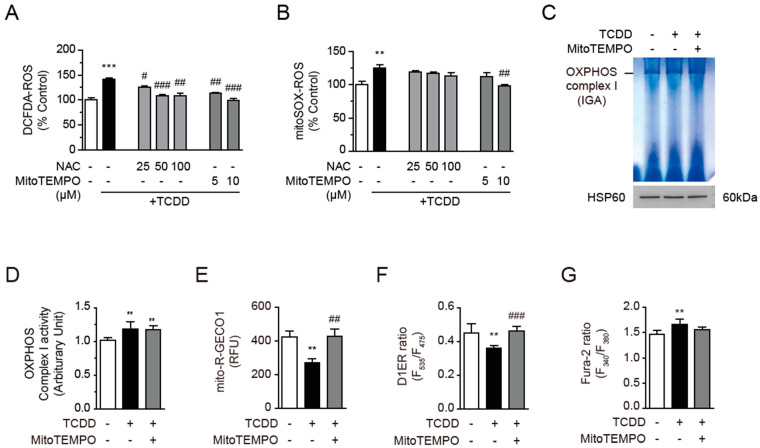
MitoTEMPO reversed the reductions in calcium in mitochondria and ER. (**A**,**B**) N-acetylcysteine (NAC) or MitoTEMPO was co-treated with TCDD (100 pM) in C2C12 cells for 24 h. Cytosolic or mitochondrial ROS were observed with a fluorometer using DCFDA (1 μM, (**A**)) or mitoSOX (5 μM, (**B**)), respectively. (**C**–**G**) C2C12 cells were incubated with TCDD (100 pM) in the presence or absence of MitoTEMPO (10 μM) for 48 h. (**C**) In-gel activity (IGA) assay of OXPHOS complex I. Isolated mitochondria from the cells were applied to blue native acrylamide gel electrophoresis, followed by an IGA assay for the OXPHOS complex I, as described in the Materials and Methods. (**D**) The HSP60 antibody in the mitochondria used in the IGA assay was analyzed via Western blot and the ratio of band intensity levels of the OXPHOS complex I to HSP60 is presented. (**E**) Mitochondrial calcium. Mito-R-GECO1-transfected cells were analyzed via confocal microscope after TCDD treatment and the fluorescent intensities are presented. (**F**) ER calcium. Calcium in the ER was measured using a fluorometer in D1ER-transfected cells. (**G**) Cytosolic calcium. C2C12 cells were loaded with Fura-2 AM (2 μM, 20 min) and changes in cytosolic calcium were monitored using a fluorometer. Data are represented as means ± SEM (*n* ≥ 4). ** *p* < 0.01, *** *p* < 0.001 vs. DMSO-treated cells; ^#^ *p* < 0.05, ^##^ *p* < 0.01, ^###^ *p* < 0.001 vs. TCDD-treated cells.

Mitochondrial ROS are generated by the OXPHOS complexes. Since TCDD increased the OXPHOS complex I activity and mtROS generation (Figure 4), we assessed whether ROS scavengers, namely N-acetylcysteine (NAC) and MitoTEMPO, reverse the action of TCDD in C2C12 cells. MitoTEMPO is a well-known mitochondria-specific superoxide scavenger and a physiochemical compound mimicking superoxide dismutase from mitochondria. It is a combination of the antioxidant piperidine nitroxide TEMPO with the lipophilic cation triphenylphosphonium, which can pass through lipid bilayers with ease and accumulate several hundred-fold in mitochondria. For instance, the inhibition of mtROS with MitoTEMPO prevented oxalate-induced injury by inhibiting mitochondrial dysfunctions [48] and reduced diabetic cardiomyopathy [49]. The co-treatment of NAC or MitoTEMPO with TCDD was efficient enough to normalize the TCDD-induced increase in DCFDA-ROS (cellular ROS) in a dose-dependent manner (Figure 7A). Only of 10 μM MitoTEMPO, but not NAC, significantly reversed the TCDD-induced increase in mitoSOX-ROS (mitochondrial ROS, mtROS) (Figure 7B). However, MitoTEMPO was unable to reverse the TCDD-mediated increase in OXPHOS complex I activity (Figure 7C,D). Instead, MitoTEMPO normalized the mitochondria and ER Ca^2+^ levels, as measured via the mitoR-GECO1 (Figure 7E) and D1ER (Figure 7F) ratios, respectively. The Fura-2 ratio (cytosolic Ca^2+^) was also normalized by MitoTEMPO (Figure 7G). These results suggest that the TCDD-mediated increase in mtROS may disturb the Ca^2+^ homeostasis, thereby reducing the mitochondrial/ER Ca^2+^ storage and insulin-triggered Ca^2+^ mobilization, while the elimination of mitochondrial ROS can restore the TCDD-induced abnormal Ca^2+^ signaling in muscle cells.

### 3.8. MitoTEMPO Protected from TCDD-Induced Insulin Resistance in the Absence of AhR

**Figure 8:**
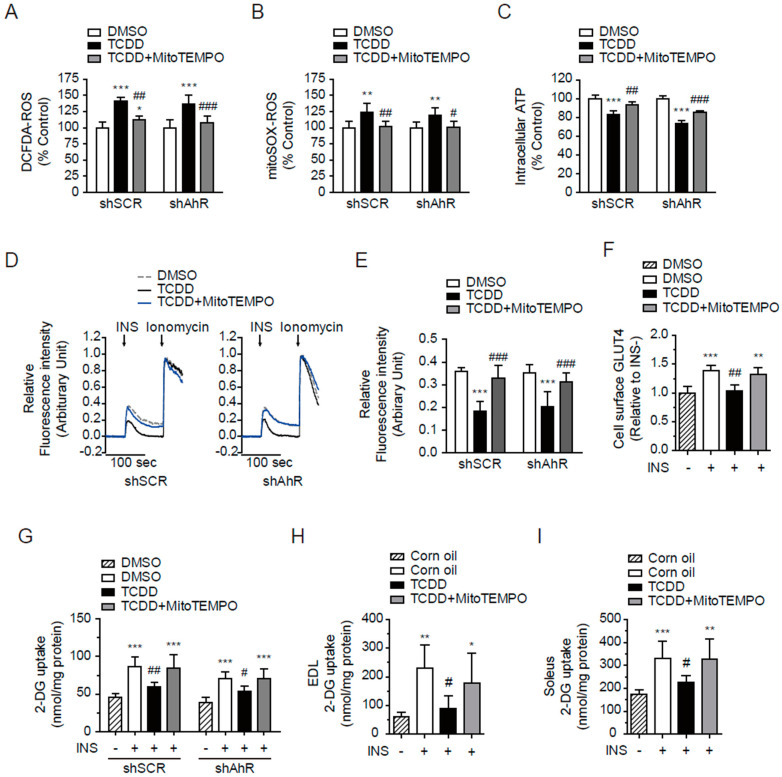
MitoTEMPO prevented TCDD-induced insulin resistance in muscles. The shSCR and shAhR C2C12 cells were incubated with 100 pM of TCDD with or without 10 μM of MitoTEMPO for 24 h in an experiment with ROS measurements and for 48 h in other experiments. (**A**,**B**) ROS generation. After the TCDD treatment, cytosolic or mitochondrial ROS were observed with a fluorometer using DCFDA (1 μM, (**A**)) or mitoSOX (5 μM, (**B**)), respectively. (**C**) The intracellular ATP contents in the cells were determined using CellTiter-Glo^®^. (**D**) The cells were loaded with 3 μM of Fluo-4 AM for 30 min and sequentially stimulated by 100 nM of insulin and 10 μM of ionomycin. The fluorescence was monitored using a confocal microscope. (**E**) The insulin-stimulated relative fluorescence intensity was calculated from (**D**). (**F**) An indirect ELISA against cell surface myc (GLUT4) was performed using the OPD reagent under non-permeabilized conditions. (**G**) Glucose uptake. The cells were incubated in glucose-free HBSS for 2 h, stimulated by insulin (100 nM, 10 min), and incubated with 2-DG (1 mM, 30 min). The concentration of 2-DG in the cells was measured using an enzymatic assay and normalized to the protein concentration. (**H**,**I**) Two weeks after the i.p. injection of TCDD and MitoTEMPO to the mice, insulin was injected 10 min before the 2-DG injection. After 30 min, the extensor digitorum longus (EDL, (**H**)) and soleus (SOL, (**I**)) muscles were dissected and the concentration of 2-DG in the muscles was measured using an enzymatic assay and normalized to protein concentration. Data are represented as means ± SEM (*n* = 4~9). Note: * *p* < 0.05, ** *p* < 0.01, *** *p* < 0.001 vs. INS- control; ^#^ *p* < 0.05, ^##^ *p* < 0.01, ^###^ *p* < 0.001 vs. TCDD alone or TCDD + INS group in cells or mice.

We further investigated whether excessive mtROS levels were related with the TCDD-induced insulin resistance. MitoTEMPO prevented the TCDD-induced production of cytosolic ROS (Figure 8A) and mtROS (Figure 8B) in both shSCR- and shAhR-C2C12 cells. MitoTEMPO also prevented the low-dose TCDD effects on intracellular ATP (Figure 8C), insulin-stimulated Ca^2+^ release (Figure 8D,E), GLUT4 translocation to the cell surface (Figure 8F), and finally glucose uptake (Figure 8G), even in the absence of AhR. In addition, mice injected with low-dose TCDD (10 μg/kg, i.p.) developed mild systemic insulin resistance as determined by an oral glucose tolerance test without altering the expression levels of IRβ, IRS, Akt, pAkt, and Glut 4 in the muscles (data not shown). However, in this model, TCDD also suppressed the insulin-stimulated glucose uptake in both EDL white (Figure 8H) and soleus red (Figure 8H) muscles. The co-injection of MitoTEMPO (10 μg/kg, i.p.) ameliorated the insulin-triggered glucose uptake in the EDL or soleus muscles of TCDD-injected mice (Figure 8H,I).

## 4. Discussion

Our current study demonstrated that insulin resistance was induced under the influence of low-dose TCDD stress in C2C12 myocytes. The TCDD treatment (100 pM) inhibited the insulin-stimulated glucose uptake due to mitochondrial defects, ROS generation, and reduced insulin-stimulated Ca^2+^ release. Unexpectedly, these low-dose TCDD-mediated effects in the muscle cells were independent of AhR and were reversed by MitoTEMPO, an mtROS scavenger.

Mitochondria are highly dynamic multifunctional organelles that are involved in a number of vital processes, such as ATP production through OXPHOS and substrate oxidation through the tricarboxylic acid (TCA) cycle and β-oxidation, the generation and detoxification of ROS, and the regulation of cellular Ca^2+^ homeostasis and apoptosis. Thus, the broad spectrum of mitochondrial features refers to the resting mitochondrial OXPHOS capacity, dynamics, turnover, and plasticity [26]. The interaction between mitochondria and insulin sensitivity is bidirectional and highly tissue-specific. Mitochondrial dysfunction, particularly in insulin-responsive tissues, may interfere with the insulin signaling pathway [13,42], in part through ROS generation [50]. Previously, we showed that an oxidized low-density lipoprotein (oxLDL) treatment reduced both the mitochondrial activity and Akt phosphorylation in A10 rat vascular smooth muscle cells (VSMC) [51]. Although the causal relationship between insulin resistance and impaired muscle mitochondrial oxidative capacity remains controversial [26], diminished mitochondrial activity was observed in insulin-resistant human skeletal muscle [17], and conversely muscle mitochondrial dysfunction resulted in insulin resistance and T2D [43].

The present study showed that low-dose TCDD induced only a slight decrease in mitochondrial activity (Figure 4), but clearly inhibited insulin-stimulated GLUT4 translocation and glucose uptake (Figure 1). Remarkably, low-dose TCDD did not significantly alter the phosphorylation of IRS-1 and Akt in insulin transduction, despite the reduced expression levels of IRβ and IRS-1 in C2C12 cells (Figure 2). There was a similar report showing that 2-aminoethoxydiphenyl borate, an inhibitor of IP_3_R and transient receptor potential (TRP) channels, led to inhibited Ca^2+^ influx and decreased insulin-induced glucose uptake without altering the Akt phosphorylation in the skeletal muscle [52]. The activation of Ca^2+^ sensing proteins following an increase in intracellular Ca^2+^ levels has been reported to directly or indirectly regulate GLUT4 exocytosis independently of the canonical insulin-Akt-GLUT4 translocation pathway [27]. In both myocytes (Figure 1) and whole muscles (Figure 8), the low-dose TCDD treatment reduced the insulin-stimulated glucose uptake by lowering the ER- and mitochondria-stored Ca^2+^ levels and abrogating the insulin-triggered Ca^2+^ efflux from the ER to the cytoplasm (Figure 5). These results led us to conclude that low-dose TCDD induces muscle insulin resistance by reducing Ca^2+^-mediated glucose uptake rather than Akt-mediated glucose uptake in the phosphorylation cascade.

There is a report showing that different second messengers were used in insulin- and contraction-mediated GLUT4 protein membrane translocation in skeletal muscle: the Ca^2+^ signals involved in insulin-mediated glucose uptake use IP_3_ as major Ca^2+^ second messengers, whereas Ca^2+^ signals for contraction-induced glucose uptake use cyclic ADP-ribose [53]. Our data suggest that TCDD may induce an insulin-resistant state in the skeletal muscle by affecting the IP_3_ or IP_3_R/RyR, because TCDD suppressed the insulin-stimulated Ca^2+^ efflux from the ER.

The intracellular Ca^2+^ levels should be tightly controlled in response to the demands of cells. This regulation relies on a series of Ca^2+^ channels, transporters, and exchangers located in the plasma membrane, the ER, and the mitochondrial membranes. The dysregulation of Ca^2+^ homeostasis is related to metabolic diseases such as obesity, insulin resistance, and T2D [54], and the intracellular Ca^2+^ levels are important for optimal insulin signaling in skeletal muscle. Biwas et al. [55] showed that 10 nM of TCDD increased the expression of RyR, a calcium channel located on the SR/ER membrane that mediates the Ca^2+^ efflux from the SR/ER to the cytoplasm. The C2C12 cells treated with 10 nM of TCDD displayed resistance to apoptotic stimuli and promoted tumor progression. In neuronal SH-SY5Y cells [56] and INS-1E β-cells [57], aberrant increases in cytosolic Ca^2+^ levels mediated by TCDD at 250 nM and 25 nM, respectively, caused cell death. Our present study showed that chronic treatment with a relatively very low concentration of TCDD at 100 pM led to increased cytosolic calcium and seems to be related with decreased Ca^2+^ levels stored in the ER and mitochondria, which is thought to result in a reduction in the insulin-triggered Ca^2+^ release from the organelles (Figure 5).

Belinger et al. [58] reported that the dysregulation of the SR Ca^2+^ release is associated with impaired muscle function as an underlying mechanism of muscle fatigue. Defective SR Ca^2+^ release and maladaptive modifications of the RyR1 have been implicated in age-dependent muscle weaknesses such as sarcopenia [59]. When RyR1 is oxidized, hyperactive Cys residues in RyR1 undergo modifications such as phosphorylation, nitrosylation, and oxidation, resulting in “leaky” RyR1 [58,60]. Our current study is comparable to previous reports showing impaired Ca^2+^ release in aged muscle, reduced SR Ca^2+^ release in SR vesicles, and reduced caffeine-induced release of the SR Ca^2+^ store [59]. An oxidized RyR1-mediated SR Ca^2+^ leak causes mitochondrial Ca^2+^ overload and increased ROS production, which in turn exacerbate the RyR1 SR Ca^2+^ leak by oxidizing the channel. Additionally, palmitate, a saturated free fatty acid, induces ER stress and depletes the ER Ca^2+^ through mitochondrial oxidative stress in podocytes [61]. Low-dose TCDD also reduced the ER Ca^2+^ levels similarly to hydrogen peroxide and thapsigargin (Figure 5), suggesting that low-dose TCDD may induce leaky SR caused by the RyR1 modification through oxidative stress.

Contrary to these reports, low-dose TCDD decreased the mitochondrial Ca^2+^ levels (Figure 5) instead of causing mitochondrial Ca^2+^ overload, which induces apoptosis by opening mitochondrial transition pores in many tissues [62]. Mitochondria may act as cytosolic Ca^2+^ buffers by regulating the activity of Ca^2+^ channels. The ER Ca^2+^ level may control the mitochondrial Ca^2+^ level, since the ER Ca^2+^ can be transported to the mitochondria through the IP_3_R-MCU complex present in the mitochondria-associated membrane (MAM) [63], the physical contact site between the ER and mitochondria. Therefore, the TCDD-induced reduction in mitochondrial Ca^2+^ could simply be associated with decreased ER Ca^2+^. Furthermore, since mitochondrial Ca^2+^ is required for various mitochondrial functions (i.e., calcium sensitive dehydrogenases participating in TCA cycle), a reduced mitochondrial Ca^2+^ level may cause decreased mitochondrial OXPHOS activities, including ATP production.

It was surprising that low-dose TCDD hyperpolarized ΔΨ_m_ as opposed to suppressing the mitochondrial respiration functions, particularly the ATP turnover rate, respiratory capacity, and ATP production. Furthermore, ΔΨ_m_ is an electrochemical gradient generated by the OXPHOS complexes (I, III, and IV), which pump protons from the matrix into the intermembrane space. There are various sites of mtROS production [64]. The predominant route of ROS production in mitochondria is the premature leakage of electrons from complexes I, II, and III [64,65], which reduce oxygen to superoxide (O2^•−^). TCDD did not influence the expression of other OXPHOS complexes except complex I (Figure 4). The hyperpolarization of ΔΨ_m_ was also observed when HEK293 cells were chronically treated with rotenone, and the chronic inhibition of complex II, III, or IV prevented rotenone-induced ΔΨ_m_ hyperpolarization [44]. This means that unless the activities of complex II, III, or IV were changed, the hyperactivity of complex I may result in proton leakage to meet oxygen demands by accumulating protons in complex I. Therefore, in muscle cells, the hyperactive complex I resulting from the upregulation of NDUFA9 expression is thought to be responsible for mitochondrial ROS production. Additionally, mismatches in electron transport and ATP synthesis during oxidative phosphorylation may be contribute to the reduced OCR and ATP levels.

In both humans and mice, the expression levels of AhR are high in the liver, lungs, heart, and placenta but low in the kidneys and muscles [66,67]. In C2C12 cells, AhR is moderately expressed. It has been reported that TCDD treatment (10 nM) in C2C12 cells induces mitochondrial stress [55] and prevents differentiation into the myotubes [68], which appears to be independent of AhR. We found that low-dose TCDD-treated AhR-knockdown cells also showed suppressed insulin-induced glucose uptake and Ca^2+^ efflux, suggesting that the TCDD-mediated insulin resistance is also independent of AhR (Figure 6). In contrast, in liver and Hepa1c1c7 cells, the TCDD-bound AhR binds to the ATP5α1 subunit of mitochondrial F(0)F(1)-ATP synthase, leading to mitochondrial hyperpolarization and decreased ATP levels [69,70].

The enhanced mtROS induced the expression levels of antioxidant proteins such as NRF2 and NQO1 (Figure 4F). As a result, the mtROS levels were almost normal after 48 h of TCDD treatment (Figure 4E). Since mitochondrial damage already occurs, this suggests that treatment is required before the peak occurrence of mtROS. Figure 8 showed that MitoTEMPO prevented all of the damage induced in low-dose TCDD-treated muscle cells that we observed. This means that mtROS is responsible for mitochondrial damage as well as disturbed Ca^2+^ homeostasis. Although the details of the action of TCDD in mitochondrial hyperpolarization and reductions in ER calcium remains to be studied further, our results suggest that dioxin-induced mitochondrial dysfunction may interfere with calcium signaling and that the regulation of mtROS is critical for dioxin-mediated insulin resistance. Since lipophilic dioxins are difficult to remove in humans, it is fortunate that the scavenging of mtROS is sufficiently effective to correct all TCDD-mediated damage in skeletal muscles. Designing therapeutics that target mtROS would open up new therapies for many degenerative diseases, including muscle insulin resistance.

## 5. Conclusions

Low-dose TCDD stress in muscle cells upregulates NDUFA9 expression, leading to a hyperactive OXPHOS complex I without altering the activity of complex II, III, or IV. The hyperactive complex I overproduces mitochondrial ROS, followed by inhibiting the IP_3_R/RyR in the SR/ER and IP_3_R-MCU complexes in the MAM. This decreases the Ca^2+^ efflux from the SR/ER and Ca^2+^ transport into the mitochondria. The dysregulation of Ca^2+^ homeostasis suppressed the GLUT4 exocytosis to the plasma membrane, leading to reduced insulin-triggered glucose uptake and insulin resistance. The removal of mitochondrial ROS using MitoTEMPO can normalize these responses, indicating that mitochondrial oxidative stress is a novel therapeutic target for EPC-induced insulin resistance. Further studies are needed to understand the molecular mechanism by which TCDD regulates OXPHOS complex 1 activity in an AhR-independent manner in skeletal muscle.

## Figures and Tables

**Table 1:** Sequences of primers used for the real-time qRT-PCR.

Genes	Accession Number	Forward Sequence (5′→3′)	Reverse Sequence (5′→3′)
AHR	NM_013464	GTGTGCAGTTGGACTTCCCT	TGGCTGGCACTGATACATGG
CYP1A1	NM_009992	TCCGGCATTCATCCTTCGTC	ACAGTTCCCGGTCATGGTTA
MCU	NM_001033259	TGATGACGTGACGGTGGTTT	CGAACGCCATCTGGTGAGTA
PKM2	NM_011099	AGCACCTGATTGCCCGAGAG	GTGAGCACGATAATGGCCCC
LDHA	NM_010699	AATGAAGGACTTGGCGGATG	ATGACCAGCTTGGAGTTCGC
LDHB	NM_008492	TGGTGGACAGTGCCTATGAAG	CATTGAGGATGCACGGGAGA
NRF2	NM_010902	5′-TTCTCCGCTGCTCGGACTA	ATGTCTTGCCTCCAAAGGATGT
NQO1	NM_008706	TAGCCTGTAGCCAGCCCTAA	GCCTCCTTCATGGCGTAGTT
18S rRNA	NR_003278	GAGCGAAAGCATTTGCCAAG	GGCATCGTTTATGGTCGGAA

## Data Availability

The data are contained within this article.

## Supplementary Materials

The following supporting information can be downloaded at: https://www.mdpi.com/article/10.3390/antiox11112109/s1. Figure S1: TCDD-induced glycolytic phenotype of muscle cells. Table S1: The sources and working dilutions of the antibodies used.
