# Supplementary material for: Low-Dose Dioxin Reduced Glucose Uptake in C2C12 Myocytes: The Role of Mitochondrial Oxidative Stress and Insulin-Dependent Calcium Mobilization
Source: Antioxidants (Basel). 2022 Oct 26;11(11):2109. doi: 10.3390/antiox11112109 (PMC9686767; doi:10.3390/antiox11112109)
Supplement: Supplementary file 1 [file antioxidants-11-02109-s001.zip › antioxidants-1918790-supplementary.pdf]

Supplementary Materials

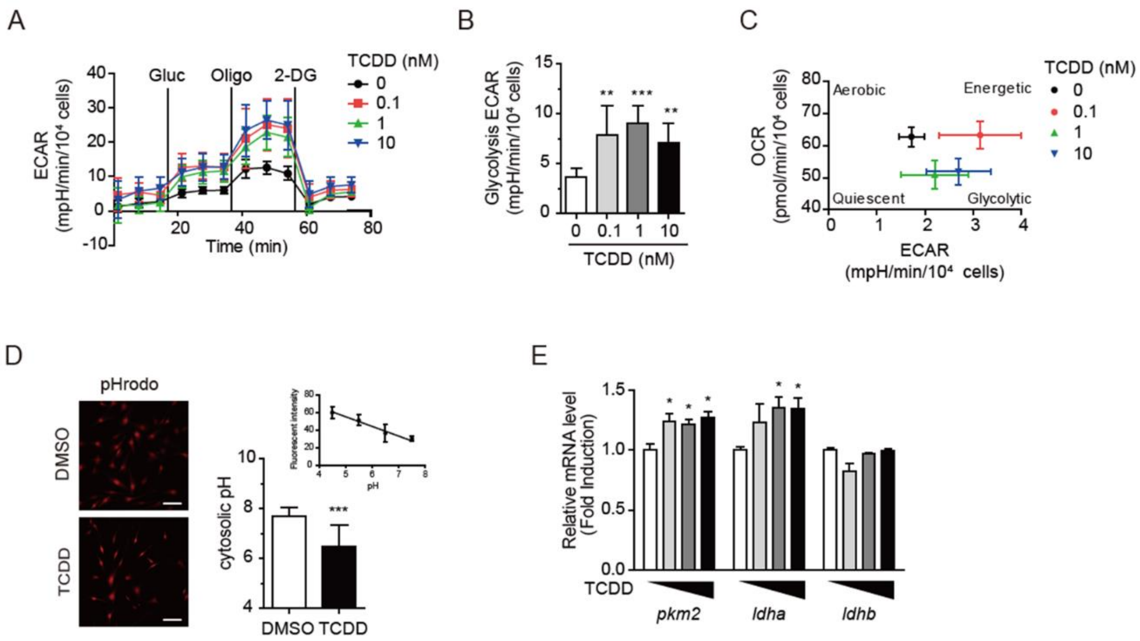

Supplementary Figure S1. TCDD-induced glycolytic phenotype of muscle cells.

(A~C) Glycolysis stress test. C2C12 cells were incubated with TCDD (0, 0.1, 1, 10 nM) for 48 h. (A) Extracellular acidification rate (ECAR) profile. ECAR was analyzed using a Seahorse XF24 analyzer. Glucose (Gluc), Oligomycin (Oligo), and 2-deoxyglucose (2-DG) were consecutively injected to measure glycolytic function. (B) Changes in ECAR caused by glycolysis after adding 10 mM glucose are represented (glucose-induced ECAR – glucose-free ECAR). (C) Metabolic profile. OCRs and ECARs under basal conditions of Fig. 3A are plotted. (D) Cytosolic pH was measured using pHrodo. C2C12 cells were stained by pHrodo Red after incubation with DMSO or 100 pM TCDD for 48 h. The intensities from confocal microscope images (left panel) were calculated as cytosolic pH (lower right) according to the standard curve (upper right) ($n \geq 19$). (E) Real-time qRT-PCR. The mRNA levels of pyruvate kinase (PKM2) and lactate dehydrogenase (LDHA and LDHB) relative to 18S rRNA depending on TCDD concentrations. The data are plotted as the mean ± SEM ($n \geq 3$). *$p < 0.05$, **$p < 0.01$, ***$p < 0.001$ vs. DMSO control.

Supplementary Table S1. The sources and working dilutions of antibodies used.

Target	Vendor	Cat. No.	Dilution
AhR	Enzo Life Sciences	BML-SA210	1:1000
Akt	Santa Cruz Biotechnology	sc-81434	1:2000
pAkt (S473)	Santa Cruz Biotechnology	sc-514032	1:2000
pAkt (T308)	Santa Cruz Biotechnology	sc-271966	1:2000
GLUT4	Novus Biologicals	NBP2-22214	1:2000
IRS-1	Cell Signaling Technology	2382S	1:2000
pIRS-1 (S307)	Santa Cruz Biotechnology	sc-33956	1:1000
pIRS-1 (Y632)	Santa Cruz Biotechnology	sc-17196-R	1:1000
IRβ	GeneTex	GTX101136	1:2000
myc	Santa Cruz Biotechnology	sc-40	1:250
NDUFA9	Invitrogen	459100	1:3000
SDHA	Abcam	ab110410	1:3000
UQCRC2	Abcam	ab14745	1:3000
COXIV	Invitrogen	A21348	1:3000
ATP5α	Abcam	ab110410	1:3000
β-actin	Santa Cruz Biotechnology	sc-47778	1:3000

Supplementary Methods

Glycolysis stress test

Glycolytic phenotype of C2C12 cells were analyzed from extracellular acidification rate (ECAR) profile under glycolysis stress as reported [36]. ECAR is primarily a measure of lactate production which can be equal to glycolytic rate. C2C12 cells (1×10^4 cells/well) were seeded on XF-24 cell culture plate (Agilent Technologies) and incubated with glucose-free XF Base Medium (Agilent Technologies) at 37°C in a non-CO_2 incubator for an hour before the experiment. During the experiment, Glucose (10 mM), Oligomycin (1 μM) and 2-DG (50 mM) were sequentially added to measure glycolysis, glycolytic capacity and reserve, respectively. ECAR was measured by XF-24 analyzer (Agilent Technologies, Santa Clara, CA, USA) and the changes in ECAR caused by addition of glucose ($ECAR_{glucose} - ECAR_{basal}$) were used to compare glycolytic activity.

Measurement of intracellular pH

Intracellular pH was monitored using pHrodo™ Red (Molecular Probes, Eugene, OR, USA) as manufacturer's instruction. C2C12 cells (1×10^4 cells) were incubated with DMSO or 100 pM TCDD for 48 h on confocal dishes. At a day of experiment, the cells were washed with Hank's Balanced Salt Solution (HBSS; 20 mM HEPES pH 7.4, 145 mM NaCl, 5 mM KCl, 1 mM $MgCl_2$, 2 mM $CaCl_2$, 5 mM D-glucose) and loaded with pHrodo™ Red at 37°C for 30 min. Images were acquired by confocal microscope, LSM700 (Carl Zeiss, Oberkochen, Germany) and standard curve to calculate intracellular pH was generated using Valinomycin (10 μM) and Nigericin (10 μM), which helps equilibrate the pH inside and outside of the cells, in pH calibration buffer (pH 4.5 – 7.5).
